# Mammalian sterile 20-like kinase 1 acts as a candidate biomarker of mortality of emergency surgical repair for acute type a aortic dissection

**DOI:** 10.1186/s12872-023-03144-8

**Published:** 2023-04-10

**Authors:** Xiaohui He, Jing Wang, Yuan Xue, Shipan Wang, Yanjun Dong, Hongjia Zhang, Meili Wang

**Affiliations:** 1 Department of Cardiac Surgery, Beijing Anzhen Hospital, Capital Medical University, Beijing, China; 2 Beijing Institute of Heart, Lung and Blood Vessel Diseases, Beijing Anzhen Hospital, Capital Medical University, Beijing, China; 3 Beijing Lab for Cardiovascular Precision Medicine, Beijing, China; 4 College of Veterinary Medicine, China Agricultural University, Beijing, China; 5 Department of Physiology and Pathophysiology, School of Basic Medical Sciences, Capital Medical University, Beijing, China

**Keywords:** Biomarker, Acute type A aortic dissection, Mammalian sterile 20-like kinase 1, 30-day mortality, Peripheral blood

## Abstract

**Background:**

Acute type A aortic dissection (ATAAD) is a life-threatening pathological change of the aorta. Patients who have undergone aortic surgery are usually at high risk of mortality.

**Aim:**

We investigated the predictive value of serum Mammalian sterile 20-like kinase 1 (MST1) as a biomarker for the risk of mortality of ATAAD patients.

**Methods:**

In this retrospective cohort study, we analyzed 160 consecutive ATAAD patients who had undergone emergency surgery from July 2016 to April 2017. Medical records and blood samples were collected and analyzed. ELISA assays were performed to detect the concentrations of several proteins including MST1. The relationship between these potential biomarkers and the primary endpoint of death was evaluated using Cox proportional hazard regression analysis.

**Results:**

Compared with a low level (< 1330.8 ng/L), high serum MST1 level (≥ 1330.8 ng/L) was positively associated with the 30-day mortality (OR = 5.233, 95%CI, 1.843–14.862, *P* < 0.01) and retained predictive after adjustment for sex, age, BMI, nasopharyngeal temperature and deep hypothermia circulatory arrest time (OR = 4.628 95% CI, 1.572–13.625, *P* < 0.01). A pre-existing basic clinical prediction model was improved with the inclusion of preoperative serum MST1. Specifically, the area under the ROC curve for base model (history of cerebrovascular disease, creatinine, time of operation) was 0.708 (95%CI, 0.546–0.836) and markedly increased to 0.823 when taking MST1 into consideration (95%CI, 0.700–0.912, *P* = 0.02).

**Conclusion:**

Our study suggests that high preoperative circulating MST1, with a concentration greater than 1330.8 ng/L, was correlated with the 30-day mortality of ATAAD patients who underwent emergency surgery.

**Supplementary Information:**

The online version contains supplementary material available at 10.1186/s12872-023-03144-8.

## Introduction

Aortic dissection refers to the pathology of blood flowing into the media of the aortic wall, ultimately leading to rupture of the aorta [1]. With the continuous improvement of surgical methods and emergency medical services, the short-term survival rate has improved for patients with aortic dissection. Despite this, there is still a high peri-operative and postoperative mortality rate. Investigations of serum biomarkers may provide opportunities for the prediction and reduction of postoperative death of patients with acute type A aortic dissection (ATAAD) and the guidance of the timing of emergency surgery.

Investigators have focused on aortic dissection biomarkers for providing fast and cost-effective methods for early diagnosis [2–6]. However, there are relatively few investigations of the relationship between biomarkers and the risk of postoperative death for ATAAD, and the predictive efficacy of biomarkers is still uncertain. New advances in understanding the risk of postoperative death for ATAAD are likely to provide innovations in preventive and therapeutic techniques.

Mammalian sterile 20-like kinase 1 (MST1) is a member of the Sterile 20 protein family. MST1 is a homologue of the Drosophila Hippo protein kinase and the core component of the Hippo signaling pathway that regulates cell division and apoptosis [7–9]. MST1 acts as a key bridge builder for apoptosis of damaged tissue. Apoptosis of vascular smooth muscle cells (VSMC) in the middle layer of the aorta is the main cause of aortic dissection [10]. When aortic dissection occurs, blood flow enters the middle layer of the blood vessels, triggers an inflammatory response and activates MST1 to promote apoptosis [11]. Yes-associated protein (YAP) is another core member of the Hippo pathway that regulates apoptosis [12, 13]. YAP is regulated by MST1 through a series of kinase cascades. All the above studies show that MST1 is critical to determining the cell fate, which plays important role in the formation and development of aortic dissection. Thus, we hypothesized that MST1 may also correlates with the postoperative mortality of ATAAD patients. In this study, we determined the relationship between the serum concentration of MST1 and the postoperative mortality of ATAAD patients.

## Methods

### Patient selection and methods

This study conformed to the principles outlined in the Declaration of Helsinki, and the Ethics Committee of Beijing Anzhen Hospital approved the study (Institutional Review Board File 2,014,019). Consecutive patients admitted to Anzhen Hospital from July 2016 to April 2017 were enrolled. The exclusion criteria were as follows: (1) Patients with a genetic syndrome related to aortic disease, such as Marfan, Loeys–Dietz, Turner, or Ehlers–Danlos syndrome, (2) Patients with traumatic dissection, inflammatory aortic disease, or congenital anomaly, and (3) Patients with a history of ascending aortic surgery (such as Bentall, David or Wheat). Ultimately, 160 ATAAD patients were enrolled into the analysis. All ATAAD patients received surgery within 48 h of admission.

The surgical procedures included 69 patients with Bentall [48 patients with TAR (thoracic aortic replacement) + SET (total arch replacement using a tetrafurcate graft and stented elephant trunk implantation) [14], 2 patients with TAR + SET + CABG (coronary artery bypass grafting), 3 patient with TAR + SET + MVR (mitral valve replacement), 1 patient with TAR + SET + TVP (tricuspid valvuloplasty), 7 patients with hemiarch replacement, 1 patient with hemiarch replacement + CABG, and 1 patient with hemiarch replacement + MVR]. Ninety-one patients had ascending aortic replacement [72 patients with TAR + SET, 4 patients with TAR + SET + CABG, 10 patients with hemiarch replacement, and 2 patients with hemiarch replacement + CABG]. The right axillary artery was used for antegrade selective cerebral perfusion when performing total arch replacement using a tetrafurcate graft and stented elephant trunk implantation under deep hypothermia circulation arrest.

### Data collection

Clinical data were assembled from the database of “A study of the prediction and treatment of Acute Aortic Syndrome (ChiCTR1900022637).” Preoperative variables were age, BMI, sex, hypertension, diabetes, respiratory diseases, history of cerebrovascular disease, history of cardiovascular disease, coronary heart disease, TEVAR, preoperative serum creatinine, ascending aorta diameter, ejection fraction, pericardial effusion, aortic regurgitation, intraoperative variables (operation time, cardiopulmonary bypass time, aortic cross-clamp time, nasopharyngeal temperature and deep hypothermia circulatory arrest time) and postoperative outcome variable (postoperative AKI, dialysis, 30-day death and late death).

### End points and definitions

The study’s primary end points were 30-day death and late death. Thirty-day mortality was defined as death from any cause during the first 30 days after surgery or as intra-hospital death after surgery. All patients were confirmed by computed tomography angiography to have type A aortic dissection (TAAD). Acute TAAD was defined as dissection involving the ascending aorta with symptoms appearing for less than 14 days [15]. Respiratory diseases include airways diseases, lung parenchymal diseases and pulmonary vascular diseases. Postoperative acute kidney injury was diagnosed according to the newest consensus-based KDIGO criteria [16]. Follow-up information was obtained from records of clinical encounters or telephone interview with patients and/or relatives.

### Blood sample analysis

After collection, blood was anticoagulated with sodium citrate and centrifuged for 15 min at 3,500 rpm at 4◦C. All samples were divided into 0.5 ml per centrifuge tube, stored at -80◦C, and used only for this study. The mean sample storage duration was 84.5 days before performing ELISA. The biomarkers assayed preoperatively by ELISA (Uscn, Wuhan, China) were Transcriptional coactivator with PDZ-binding motif (TAZ), Yes-associated protein (YAP1/2), Mammalian sterile 20-like kinase (MST1/2), and Large tumor suppressor kinase (LATS1/2) [17] . All ELISA experiments were performed twice, and the mean value was used for analysis.

### Statistical analysis

Data were expressed as frequency and percent, as mean ± standard deviation, or as median with interquartile range. We used the Pearson’s χ^2^ test or Fischer’s exact tests (in case of expected values < 5) for categorical variables, one-way ANOVA for normal continuous variables, and the Kruskal–Wallis test for skewed continuous variables. Patients were then stratified into two groups comparing the upper quartile (“Q3”, *n* = 54) with the lower two quartiles (“Q1–2”, *n* = 106) Cox proportional hazard regression analysis was performed to identify the predictors of 30-day and late mortality. Both unadjusted and multivariate-adjusted regression models were used to evaluate the association of a biomarker with mortality after surgery. Because the number of end-point events was limited, we present regression results only as (1) an unadjusted model, (2) a model adjusted for age and sex, and (3) a model adjusted for age, sex, BMI, nasopharyngeal temperature and deep hypothermia circulatory arrest time. The potential for the assessed biomarkers to diagnose 30-day mortality was further examined using receiver operating characteristic (ROC) curves generated for the models. The difference in area under the curve (AUC) of the analyses was assessed using DeLong test for paired ROC curves. The additive benefit of considering the biomarker in diagnosing 30-day mortality was assessed in sensitivity analyses that compared the AUC of a base model (only history of cerebrovascular disease, creatinine, time of operation) to a model that included biomarker and the aforesaid covariates. We calculated the survival rate using the Kaplan–Meier analytical method; patients were categorized into tertiles based on the corresponding biomarker, whereas differences between groups were evaluated using the log-rank test. All tests were 2-sided and differences were considered statistically significant at *P*- value < 0.05. All analyses were performed using statistical software package R (http://www.R-project.org, The R Foundation).

## Results

### Biomarkers and primary outcomes of ATAAD patients

Table 1 shows the circulating concentrations of potential biomarkers. No differences in preoperative plasma concentrations of TAZ, YAP1, YAP2, MST1, LATS-1 and LATS-2 were observed for either group. However, we found preoperative MST1 was higher in patients who died within 30 days. Patients in the Outcome Cohort were stratified into two groups (Q1-2, Q3) (Table 2).

**Table 1:** Biomarkers of participants in ATAAD

	No 30-day death	30-day death	P-value	No Death	Death	*P*-value
N	143	17		128	32	
TAZ, ng/ml	14.3 ± 3.0	14.0 ± 2.5	0.656	14.5 ± 3.0	13.6 ± 2.4	0.151
YAP 1, ng/ml	11.3 ± 2.6	11.3 ± 2.7	0.915	11.2 ± 2.6	11.9 ± 2.8	0.140
YAP 2, ng/ml	10.8 ± 2.6	10.8 ± 3.0	0.981	10.7 ± 2.5	11.1 ± 3.1	0.384
MST 1, pg/ml	1152.4 ± 255.7	1386.6 ± 139.3	< 0.001*	1157.0 ± 252.5	1258.3 ± 258.5	0.045*
MST 2, pg/ml	1108.2 ± 242.0	1145.3 ± 245.1	0.551	1115.6 ± 243.1	1098.2 ± 240.1	0.718
LATS 1, ng/ml	9.1 ± 1.9	8.2 ± 1.8	0.070	9.1 ± 1.9	8.6 ± 1.8	0.159
LATS 2, ng/ml	8.6 ± 1.7	8.8 ± 1.9	0.677	8.7 ± 1.7	8.5 ± 1.7	0.440

^*^P value < 0.05

**Table 2:** Baseline characteristics of participants in ATAAD

	Total	Q1–Q2 (< 1330.8 ng/L)	Q3 (≥ 1330.8 ng/L)	*P*-value
N	160	106	54	
Age, years	49.2 ± 11.5	48.4 ± 11.4	50.7 ± 11.6	0.237
Sex (male)	118 (73.8%)	83 (78.3%)	35 (64.8%)	0.067
BMI, kg/m^2^	25.9 ± 3.7	26.0 ± 3.8	25.8 ± 3.7	0.741
Hypertension	106 (66.2%)	70 (66.0%)	36 (66.7%)	0.937
Smoking	66 (41.2%)	41 (38.7%)	25 (46.3%)	0.355
Diabetes	10 (6.2%)	6 (5.7%)	4 (7.4%)	0.666
Respiratory diseases	3 (1.9%)	2 (1.9%)	1 (1.9%)	0.988
History of cardiovascular disease	20 (12.5%)	13 (12.3%)	7 (13.0%)	0.899
History of cerebrovascular disease	8 (5.0%)	4 (3.8%)	4 (7.4%)	0.319
Coronary heart disease	9 (5.6%)	7 (6.6%)	2 (3.7%)	0.452
TEVAR	5 (3.1%)	3 (2.8%)	2 (3.7%)	0.764
Creatinine, μmol/L	78.8 (67.2–97.3)	79.0 (66.6–97.1)	78.7 (67.5–100.9)	0.602
White blood cell, 10^9^/L	10.4 ± 3.8	10.2 ± 3.7	10.7 ± 4.0	0.447
Platelet, 10^9^/L	211.6 ± 74.0	211.3 ± 76.2	212.2 ± 70.0	0.939
Troponin I, ng/mL	0.0 (0.0–0.0)	0.0 (0.0–0.1)	0.0 (0.0–0.0)	0.514
Myoglobin, ng/ml	27.9 (16.4–58.9)	26.7 (16.1–46.4)	34.2 (18.6–66.8)	0.150
Fibrinogen, g/L	3.6 ± 1.8	3.8 ± 2.0	3.3 ± 1.4	0.148
D-dimer, ng/mL	1137.0 (563.0–2993.0)	1563.0 (589.5–3028.5)	1067.0 (444.8–2734.2)	0.955
Ascending aorta diameter, mm	46.9 ± 8.5	46.9 ± 8.8	43.0 ± 8.8	0.912
Ejection fraction, %	61.7 ± 6.4	62.3 ± 6.2	60.5 ± 6.5	0.087
Pericardial effusion	34 (21.2%)	21 (19.8%)	13 (24.1%)	0.533
Aortic regurgitation				0.457
Mild	53 (33.1%)	31 (29.2%)	22 (40.7%)	
Moderate	36 (22.5%)	25 (23.6%)	11 (20.4%)	
Severe	36 (22.5%)	24 (22.6%)	12 (22.2%)	
Time of operation, hour	7.5 ± 1.7	7.3 ± 1.6	7.7 ± 1.9	0.135
Aortic crossclamp time, min	114.7 ± 37.8	114.4 ± 37.3	115.4 ± 39.2	0.882
Nasopharyngeal temperature,℃	24.5 ± 2.3	24.8 ± 2.4	24.1 ± 1.9	0.090
Deep hypothermia circulatory arrest	136 (85.0%)	89 (84.0%)	47 (87.0%)	0.607
Deep hypothermia circulatory arrest time, min	21.2 ± 12.2	20.9 ± 12.5	21.8 ± 11.6	0.664
30-day death	17 (10.6%)	5 (4.7%)	12 (22.2%)	< 0.001
AKI	91 (57.2%)	59 (55.7%)	32 (60.4%)	0.571
Neurological complications	19 (11.9%)	14 (13.2%)	5 (9.3%)	0.465
Dialysis	17 (10.6%)	8 (7.5%)	9 (16.7%)	0.077

Results are expressed as n (%) or mean ± standard deviation (SD) or median interquartile range (IQR)

*BMI* Body mass index, *TEVAR* Thoracic aortic endovascular repair, *AKI* Acute kidney injury

^*^P value < 0.05

### Baseline characteristics of ATAAD patients

Table 2 displays the demographics and baseline characteristics of the entire 160-patient study population. The mean age in the cohort was 49.2 ± 11.5 years, and 118 participants were men (73.8%). Among them, 17 (10.6%) patients died within 30 days after surgery. There were no difference in the patient characteristics or intraoperative details between the two groups.

### Serum MST1 was associated with clinical outcome

Table 3 demonstrates that MST1 was significantly associated with the 30-day mortality (OR = 1.004, 95% CI, 1.002–1.007, *P* < 0.01). After adjustment for age, sex, and BMI, the OR was 1.004 (95% CI, 1.001–1.006, *P* < 0.01). Of note, there was statistically significant difference regarding the 30-day mortality-associated factors between the “Q1–2” and “Q3” group (OR = 4.628, 95% CI, 1.572–13.625, *P* < 0.01). Table 3 also suggests that MST1 was significantly associated with the mortality after follow-up (*P* = 0.032). Nevertheless, statistical significance was not reached after adjusting for age, sex, BMI, nasopharyngeal temperature and deep hypothermia circulatory arrest time (*P* = 0.105).

**Table 3:** Cox-Regression Analyses for MST1 and the 30-day death and long-term survival

Exposure	Non-adjusted OR (95% CI) *P*-value	Adjust I OR (95% CI) *P*-value	Adjust II OR (95% CI) *P*-value
**30-day death**			
MST 1 (pg/ml)			
Continuous	1.004 (1.002, 1.007) 0.001*	1.004(1.001, 1.006) 0.002*	1.004 (1.001, 1.006) 0.004*
MST 1 (pg/ml)			
Q1–Q2	Reference	Reference	Reference
Q3	5.233 (1.843, 14.862) 0.002*	5.034 (1.740, 14.566) 0.003*	4.628 (1.572, 13.625) 0.005*
**Postoperative death**			
MST 1 (pg/ml)			
Continuous	1.002 (1.000, 1.003) 0.032*	1.001 (1.000, 1.003) 0.051	1.001 (1.000, 1.003) 0.105
MST 1 (pg/ml)			
Q1–Q2	Reference	Reference	Reference
Q3	2.023 (1.010, 4.055) 0.046*	1.828 (0.897, 3.728) 0.097	1.704 (0.821, 3.538) 0.152

Non-adjusted model adjust for: None

Adjust I model adjust for: Age; Sex

Adjust II model adjust for: Age; Sex; BMI; Nasopharyngeal temperature; Deep hypothermia circulatory arrest time

^*^P value indicates significance at *P* < 0.05. *CI* Confidence interval; HR, hazards ratio

**Fig. 1:**
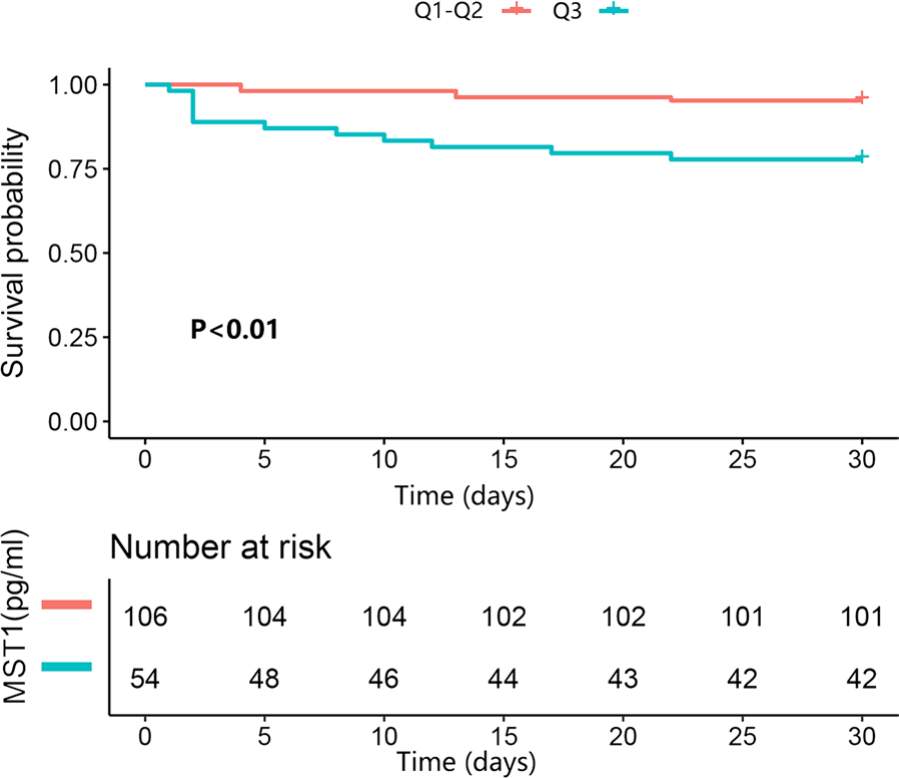
Kaplan–Meier analysis of MST1 with the outcome of 30-day mortality. Log-rank P values and number of participants within each category are shown

Kaplan–Meier curves are shown in Fig. 1, which reveals a significantly higher mortality rate of the “Q3” group compared to the “Q1–2” group (log-rank *P* < 0.01) after a median follow up period of 3.89 years.

### Incremental effects of biomarkers in addition to the traditional risk factors

**Fig. 2:**
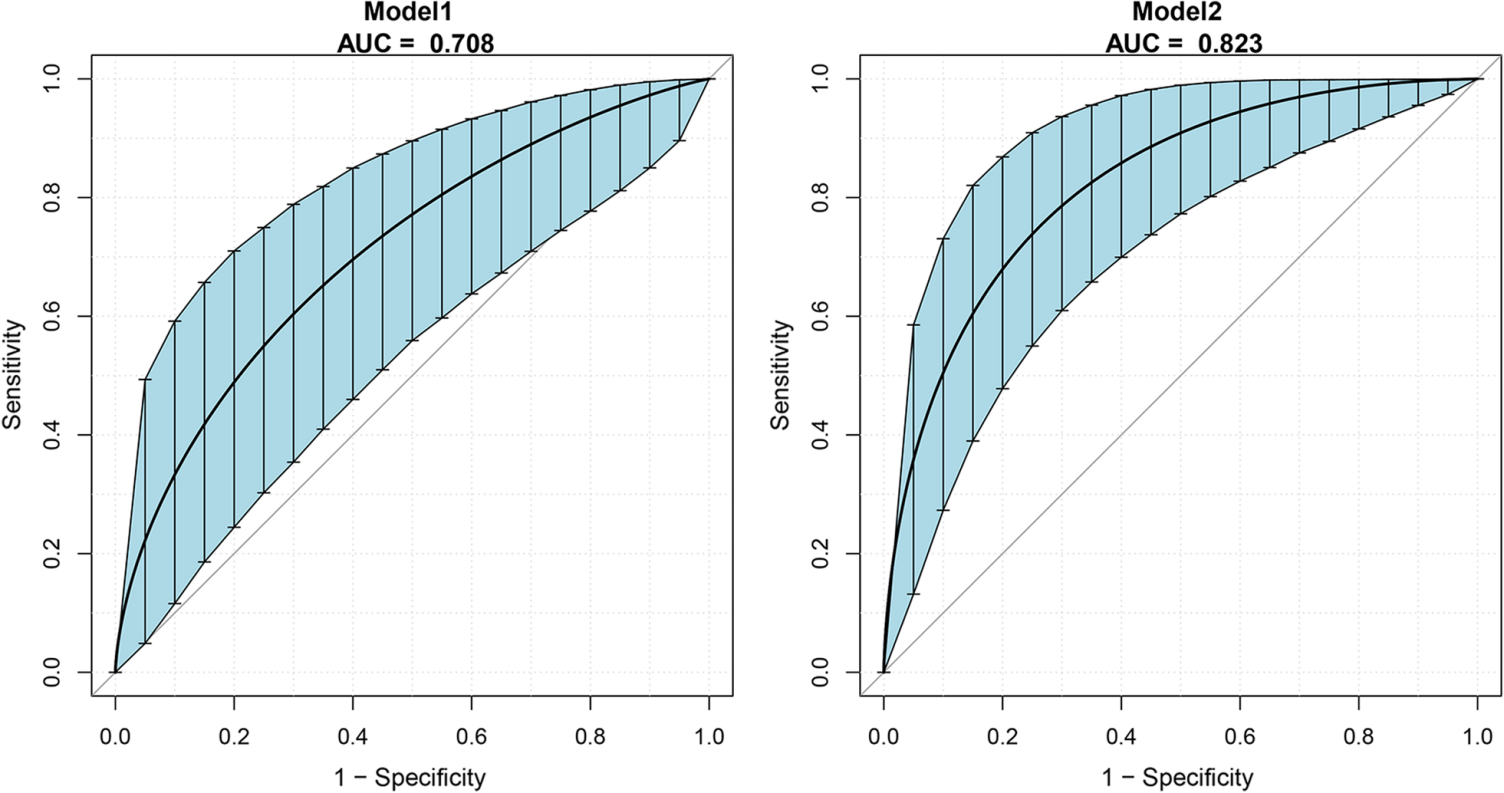
Receiver operating characteristic (ROC) curve analyses assessing the ability of preoperatively measured markers to diagnose 30-day mortality. Receiver operating characteristic curve (ROC) analyses for the prediction of 30-day death. AUC confidence interval and significance tests using Bootstrap resampling (times = 500). The blue shading denotes the bootstrap estimated 95% confidence interval with the AUC. Model 1 comprises history of cerebrovascular disease, CREA, time of operation based on differences observed between groups on recruitment (Table 1). ROC area (AUC): 0.708 (95%CI, 0.546-0.836). Model 2 comprises the MST1, history of cerebrovascular disease, CREA, time of operation. ROC area (AUC): 0.823 (95%CI, 0.700-0.912). *P* values (*P* = 0.02) relate to the comparison of Model 1 to the Model 2 using DeLong test for matched ROC curves.

The ability of preoperative MST1 concentrations to predict the 30-day mortality was investigated using ROC curves (Fig. 2). To test whether the inclusion of preoperative MST1 enhanced the predictive value of the traditional risk factor screening, we compared this basic model with models that additionally included the MST1. The AUC for the base model (which included history of cerebrovascular disease, creatinine, time of operation) was 0.708 (95%CI, 0.546–0.836) and markedly increased when considered in conjunction with MST1 (models 2: AUC, 0.823, 95%CI, 0.700–0.912, *P* = 0.02).

## Discussion

In this study, we retrospectively analyzed the association of MST1 concentration in preoperative peripheral blood with postoperative mortality in 160 ATAAD patients who had undergone surgical repair of the aorta. Promising results demonstrated that preoperative MST1 concentration was significantly correlated with the mortality within 30 days after surgery, but in the follow-up process, the correlation was unclear between MST1 concentration and the long-term mortality. Consequently, MST1 could be a new biomarker for the prediction of short-term risk of death after ATAAD surgery, and pre-procedural MST1 concentration might be helpful in identifying patients who would benefit from emergency surgery.

### MST1 is a promising factor for predicting the risk of short-term mortality

**Fig. 3:**
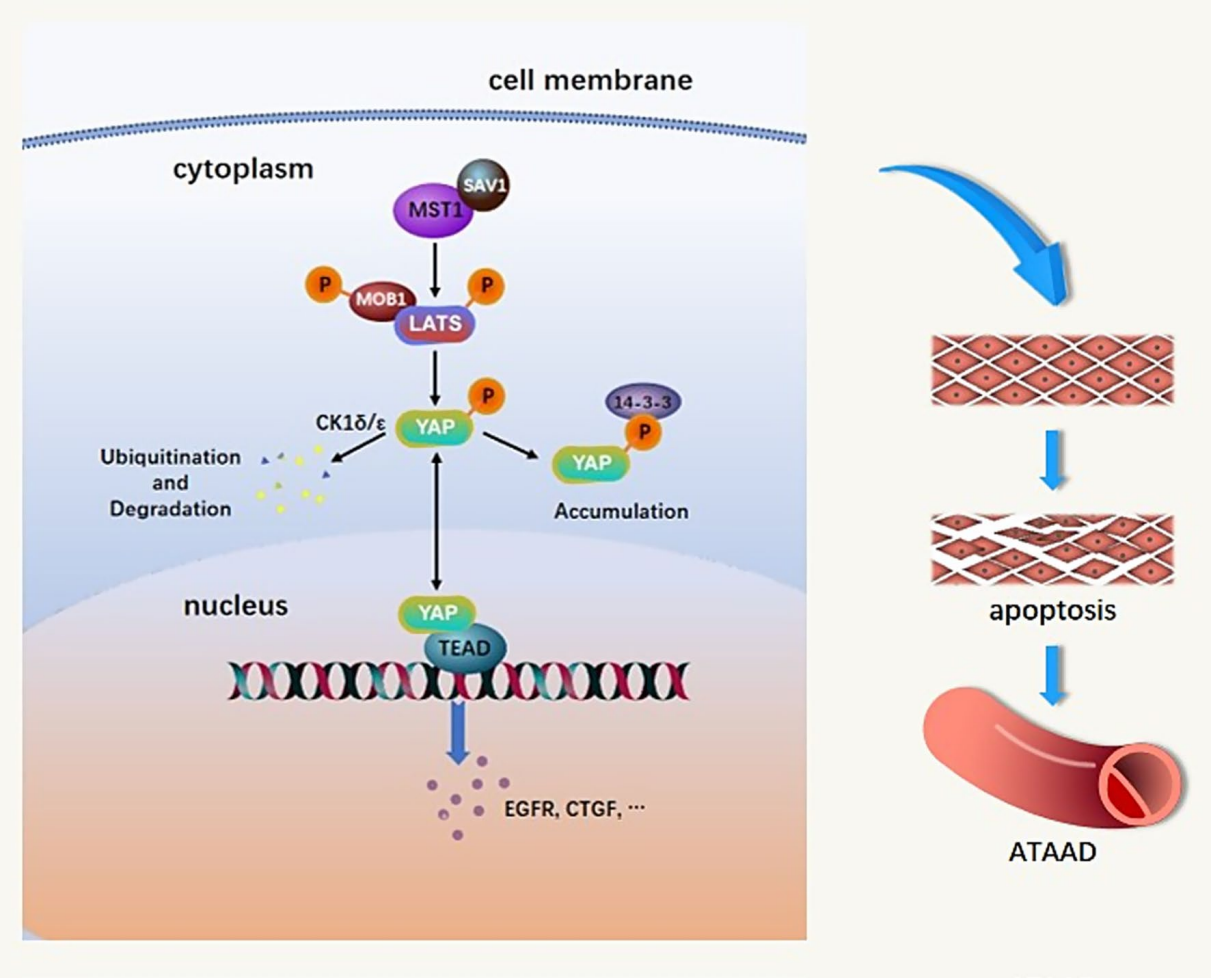
Hypothetical diagram of the mechanism of MST1 involved in the pathogenesis of ATAAD.

MST1 is widely expressed in human tissues and integral to processes such as cell differentiation, adhesion, migration, and apoptosis [18]. Regarding the working model of MST1 in the mammalian Hippo signaling pathway, it is currently accepted that MST1 plays a regulatory role in the pathway through a series of kinase cascades (Fig. 3). Mst1 kinase interacts with and phosphorylates the adaptor protein Sav1, after which they phosphorylate and activate Lats kinase and its associated protein, Mps One Binder kinase activator protein 1 (Mob1). Mob1 and Lats then phosphorylates the downstream effector YAP [19–21] . Phosphorylation of YAP induces 14–3-3 binding and cytoplasmic accumulation. Alternatively, it initiates CK1δ/ε phosphorylation, which ultimately leads to ubiquitination and degradation [22, 23]. As the major target transcription factors of YAP, members of the TEA domain transcription factor family (TEAD) are the transcription factors that most frequently interact with YAP. When YAP is dephosphorylated and enters the nucleus, it binds and activates the transcription factor TEAD, which mediates the expression of genes related to cell proliferation and anti-apoptosis [24]. In our previous study, YAP downregulation induced an increase in VSMC apoptosis induced by mechanical stress, and downregulation of MST1 leads to YAP transactivation. In addition, YAP activation stimulated the proliferation of cardiomyocytes [25, 26]. Therefore, MST1, as an important meeting point in regulating apoptosis, is crucial for determining cell fate.

It is noteworthy that the results of this study agree with the aforesaid theory. The Cox regression analysis of the detection results for each biomarker revealed that ATAAD patients whose preoperative peripheral blood contained high levels of MST1 also had greater short-term mortality after surgery. We speculated that high MST1 expression inhibited YAP transcription and increased VSMC apoptosis by inducing the phosphorylation of YAP through the Hippo pathway. For ATAAD patients, the higher the preoperative plasma level of MST1, the more severe the apoptosis of VSMC occurs, the more serious the condition. Thus, MST1 is promising as a new biomarker for preoperative screening of patients at higher risk of death from emergency surgery.

### MST1 can enhance the ability for typical independent risk factors to assess the short-term prognosis of ATAAD

The history of cerebrovascular disease, creatinine, and time of operation are independent predictors of short-term death in ATAAD [27–30]. In this study, we investigated whether the inclusion of MST1 could improve the accuracy of the basic model of the three independent factors in predicting the risk of short-term mortality in ATAAD. Compared with the basic predictive model that consisted of cerebrovascular history, creatinine and time of operation, the AUC of the ROC curve combined by four risk factors (including MST1) was significantly increased to 0.823 (95%CI, 0.7002 -0.9119) (*P* = 0.02). This evidence indicated that MST1 can enhance the ability of typical independent risk factors to assess the short-term prognosis of ATAAD. Moreover, our previous study found that the prognosis of patients with ATAAD could be significantly improved if they can survive from the acute stage safely under conservative medical treatment [31] . Thus, MST1 is promising as a biomarker for preoperative risk prediction according to this property. When surgeons find that patients are at high risk of postoperative death after comprehensive analysis of the clinical conditions, surgeons can consider whether to proceed with emergency surgery and inform patients' families, in a timely manner, the risks and benefits that accompany surgical intervention.

### Limitations

Because of a limited capacity of the detection reagent and the medical urgency, we were underpowered to assess the association of the phosphorylation of YAP with surgical prognosis or to compare the level of phosphorylated YAP from ATAAD patients with high risk of 30-day death with those whose risk of short-term death after surgery was lower. Another limitation was the lack of analysis of MST1 in tissue samples. We assessed some of the biomarkers in the peripheral blood to detect patterns between biomarkers and poor prognostic outcome, to provide a fast, cost-effective, and non-invasive method for predicting prognosis in the clinic. However, it is unclear whether the lower values of MST1 in tissues of aorta before ATAAD operations will attenuate the elevated risk for adverse events. The answer to this question will require additional studies to investigate Hippo-MST signaling pathway using aortic tissues.

Although we adjusted for sex, age, BMI, nasopharyngeal temperature and deep hypothermia circulatory arrest time in an additive model, we are aware of the possibility of residual confounding. For this reason, we could not assess accurately the value of a combined biomarker base model to predict end points. The fact that this study was conducted at a single center may have introduced undetected bias. Nevertheless, although future multicenter studies with more patients are necessary to validate fully the use of MST1 in ATAAD risk stratification, the present study is an important step towards demonstrating the value of Hippo pathway biomarkers for predicting mortality in ATAAD patients.

## Conclusion

Preoperative concentrations of MST1 greater than 1330.8 ng/L was significantly associated with the 30-day mortality in ATAAD patients who underwent surgical repair of the aorta. This association is likely related to the high MST1 expression-mediated inhibition of YAP transcription and the enhancement of VSMC apoptosis. The high serum concentration of MST1 not only reflects the ATAAD severity, but also provides beneficial application value in the assessment of emergency surgery.

## Supplementary Information

**Additional file 1**. Statement on informed consent waiver

## Data Availability

The datasets analyzed during the current study are available from the corresponding author on reasonable request.
