# Supplementary material for: Mammalian sterile 20-like kinase 1 acts as a candidate biomarker of mortality of emergency surgical repair for acute type a aortic dissection
Source: BMC Cardiovasc Disord. 2023 Apr 10;23:188. doi: 10.1186/s12872-023-03144-8 (PMC10088138; doi:10.1186/s12872-023-03144-8)
Supplement: Supplementary file 1 — Additional file 1. Statement on informed consent waiver [file 12872_2023_3144_MOESM1_ESM.docx]

**Statement on informed consent waiver**

Department of cardiac surgery Hongjia Zhang applied for the project ‘Mammalian sterile 20-like kinase 1 acts as a candidate biomarker of mortality of emergency surgical repair for acute type A aortic dissection’.

The following conditions are met:

☑ Studies using medical records and biological samples obtained from previous clinical visits (retrospective studies).

☑ Subjects' privacy and personally identifiable information is protected.

For the above reasons, informed consent is waived.

Ethics Committee of Beijing Anzhen Hospital, Capital Medical University
